# Fucoidan modulates SIRT1 and NLRP3 to alleviate hypertensive retinopathy: in vivo and in vitro insights

**DOI:** 10.1186/s12967-024-04877-6

**Published:** 2024-02-15

**Authors:** Jing Li, Xiaochen Wang, Jie Bai, Huangzhao Wei, Wenbo Wang, Shuai Wang

**Affiliations:** 1 Department of Ophthalmology, Second Affiliated Hospital of Dalian Medical University, Dalian, 116023 China; 2 Department of Public Health Experimental Teaching Center, Dalian Medical University, Dalian, 116044 China; 3 Dalian Institute of Chemical Physics, Chinese Academy of Sciences, Dalian, 116023 China

**Keywords:** Hypertensive retinopathy, SIRT1, NLRP3 inflammasome, Fucoidan therapeutic potential

## Abstract

**Background:**

Hypertension influences the inflammatory pathological changes in the retina. The function of the inflammasomes is significant. To see if Sirtuin 1 (SIRT1) regulates angiotensin II (Ang II)-induced hypertensive retinopathy and inflammation by modulating NOD-like receptor thermal protein domain associated protein 3 (NLRP3) inflammasome activation and the potential protective effects of fucoidan (FO) in mouse retinal vascular endothelial cells (mRECs) and mice retina.

**Methods:**

The diagnosis of hypertensive retinopathy was made after three weeks of Ang II infusion (3000 ng/kg/min). One day prior to the commencement of Ang II infusion, the mice were treatment with NLRP3 inhibitor MCC950 (10 mg/kg/day, intraperitoneal injections) or FO (300 mg/kg/day, oral gavage). A blood pressure was recorded. Hematoxylin and eosin (H&E) staining was used to conduct pathological alterations, dihydroethidium bromide (DHE) was utilized to assess oxidative stress damage in the retina, and fluorescence angiography was used to identify vascular disorders in the eye. Using immunohistochemical labeling, NLRP3 expression was found. Reactive protein and mRNA expression levels in mouse retina and cells were assessed using Western blot and real-time quantitative polymerase chain reaction (RT-qPCR).

**Results:**

NLRP3 inflammasome activation and SIRT1 decrease were brought about by Ang II infusion. Retinopathy and dysfunction were lessened by MCC950 target-induced NLRP3 inflammasome activation, while overexpression of SIRT1 had the opposite impact on NLRP3 inflammasome activation, indicating that SIRT1 functions as an upstream regulator of NLRP3 activity. FO may improve SIRT1 expression and decrease NLRP3 activation in retinopathy and dysfunction brought on by Ang II, and the effects were consistent across both in vivo and in vitro models.

**Conclusions:**

SIRT1 adversely regulates the NLRP3 inflammasome pathway, which in turn increases Ang II-induced inflammation and hypertensive retinopathy. FO may mitigate Ang II-induced retinopathy and dysfunction via modulating the expression of SIRT1/NLRP3. This implies practical approaches to the management of hypertensive retinopathy.

## Background

Many vision-threatening eye disorders, such as non-arteritic anterior ischemic optic neuropathy, retinal vascular occlusion, retinal macroaneurysm, and hypertensive retinopathy (HR), are associated with an increased risk of hypertension [1]. Furthermore, hypertension has been linked to the etiology of age-related macular degeneration and may worsen the vision-threatening consequences of diabetic retinopathy. Hypertensive retinopathy and choroidopathy, which are direct manifestations of chronic hypertension in the eye, are indicative of a systemic pathology that affects the whole body [2].

There is growing evidence that inflammation plays a role in the etiology of hypertension. [3]. Inflammasomes are involved in the pathophysiology of several inflammatory disorders. A sequence of protein oligomerization events that trigger the identification of certain molecular patterns from infections, cellular injury, or changed homeostatic circumstances is required for inflammasomes formation [4]. The NOD-like receptor thermal protein domain associated proteins (NLRPs), which include NLRP1, NLRP3, NLRC4, and missing in melanoma 2 (AIM2), are the four primary constituents of the inflammasomes. The most researched of which are NLRP3 inflammasomes [5, 6]. In the host’s immunological response to infections and sterile injuries, the NLRP3 inflammasome is essential [7]. One important stage in the activation of inflammasomes is the overexpression of NLRP3 [8]. Two signals are needed for the NLRP3 inflammasome to activate. Nuclear factor kappa B (NF-κB) signaling is triggered in one way [9], while certain chemicals, such as mitochondrial reactive oxygen species, may activate a second signal [10]. The activation process of cysteine protease CASP1 is carried out by the NLRP3 inflammasome, which provides a molecular platform for the release of mature CASP1 (CASP1 p20) and the production of proinflammatory cytokines, namely IL-18 and Interleukin-1β (IL-1β) [11, 12]. Even though the NLRP3 inflammasome has been studied in great detail, little is known about the endogenous mechanisms that control the NLRP3 inflammasome negatively.

Reactive oxygen species (ROS) is considered as one of the triggers of NLRP3 inflammasome activation. ROS are unstable and highly reactive molecules produced by reduction of oxygen mainly during mitochondrial oxidative phosphorylation. Excessive ROS production and/or failure of anti-oxidant defense systems result in oxidative stress leading to damage of cellular macromolecules including nucleic acids, proteins, and lipids, and has been implicated in pathogenesis of several diseases [13]. A member of the NAD + -dependent deacetylase enzyme family, sirtuin (SIRT) controls a number of cellular targets and activities. The most extensively investigated is SIRT1. Both in vitro and in vivo, SIRT1 regulates the generation and build-up of ROS [14–16]. Excessive inflammation brought on by ROS buildup results in mitochondrial malfunction and cell death [17]. According to recent research, SIRT1 may inhibit inflammatory reactions that are mediated by the NF-κB signaling pathway. Conversely, SIRT1 also activates AMP-activated protein kinase alpha 1 (AMPK), peroxisome proliferator activated receptor alpha (PPARα), and peroxisome proliferative activated receptor, gamma, coactivator 1 alpha (PGC-1α), which collectively function as inhibitors of NF-κB signaling. These actions subsequently stimulate the production of oxidative energy and mitigate inflammation [18]. However, it is uncertain whether SIRT1 could downregulate NLRP3 expression.

Due to its numerous beneficial properties, including its anti-inflammatory properties, fucoidan (FO), a fucose-enriched sulfated polysaccharide, has been widely utilized as a dietary supplement and health food [19–21]. Research indicates that the administration of FO may mitigate renal fibrosis caused by diabetes by upregulating SIRT1 protein levels via overexpression [22].

In order to highlight a unique targeted method to treat HR, we investigated the effects of FO as a protective agent on SIRT1/NLRP3 in Ang II-induced retinopathy.

## Materials and methods

### Animals

Wukong Biotechnology (Jiangsu, China) provided 40 8-week-old male C57BL/6 mice, which we used in our investigation as wild type (WT) animals. A week of adaptive feeding is required for all animals prior to experimentation. As previously mentioned, Ang II infusion (3000 ng/kg/min, aladdin, 4474–91-3) or saline infusion utilizing osmotic mini-pumps (ALZET MODEL1004, 28 days, DURECT, Cupertino, CA) for 3 weeks were used to produce hypertensive retinopathy model [23]. All of the animals were sedated when we withdrew the ocular tissues after the Ang II and saline infusion. The Institutional Animal Care and Use Committee (IACUC) of Dalian Medical University authorized all animal experiments, and the research followed the NIH's (No. 85–23; Berthesda, MD, USA) Guide for the Care and Use of Laboratory Animals.

### Inhibition of NLRP3 inflammasome in mice

The mice were administered with NLRP3 inhibitor MCC950 (10 mg/kg/day, intraperitoneal injections; HY-12815A, MedChem Express, Shanghai, PRC) in 200 μl of normal saline once daily from one day before Ang II infusion to the day of euthanasia (HR model) [24].

### Fucoidan (FO) treatment in mice

The mice were fed with FO (300 mg/kg/day, HY-132179, MedChem Express, Shanghai, PRC) in 200 μl of normal saline once daily from one day before Ang II infusion to the day of euthanasia (HR model) [25].

### Blood pressure monitoring method

The tail-cuff device monitored blood pressure. The mice were put on a fixator and allowed to adapt to a heating pad for 10 min before to the measurement. When the waveform was stable, the tail was completely exposed, and blood pressure readings were taken. At least five measurements were made of each mouse.

### Fluorescence angiography

We used an intraperitoneal injection of 2.5% tribromoethanol (0.020 mL/g; Sigma-Aldrich, Dorset, UK) to anesthetize the mice. One compound-tropicamide eye drop was used to dilate each pupil, and then the eye was treated with ophthalmic gel (hypromellose 2.5% ophthalmic-demulcent solution; Gonak; Akorn, Lake Forest, IL, USA). The mice were subsequently given a tail vein injection of fluorescein sodium (13 mL/kg in saline; Alcon, TX, USA). After that, for five minutes, we used a retinal imaging equipment (OPTO-RIS; Optoprobe Science, Burnaby, BC, Canada) to take pictures of the retinal arteries every thirty seconds. The branch architecture and pulsatile activity of arteries were used to identify them. In order to determine the arteriovenous ratio for each mouse, we selected an identifiable anatomical site that was two optic-disc diameters from the optic disc. ImageJ (Rasband; NIH) software was used to compare measurements [25].

### Histological analyses

The eye tissues were implanted in a paraffin block or OCT after being preserved with 4% paraformaldehyde for several days. The tissues from the eyes were cut into 8 μm fresh frozen sections and 4 μm paraffin sections. Dihydroethidium (DHE) staining was used to assess oxidative stress damage in the retina, while hematoxylin and eosin (H&E) staining was used to evaluate pathological alterations. Details on the DHE and H&E staining process were according the kit instructions.

### Immunohistochemical staining

Briefly, the eye sections were incubated for 10 min with hydrogen peroxide, followed by an hour at room temperature with 5% BSA closure. Next, they were incubated at 4 °C overnight with a specific primary antibody, anti-NLRP3 (1:200, ET1610-93, HUABIO). The following day, the sections were washed with PBS and incubated for one hour at room temperature with horseradish peroxidase. Subsequently, the slices were examined under a microscope after being stained with DAB solution and then again with hematoxylin.

### Cell culture and treatment

Procell (ml096624, mlbio, Shanghai, China) provided the mouse retinal vascular endothelial cells (mRECs), which were then cultivated in 89% high glucose-dulbecco's modified eagle medium (H-DMEM) + 10% fetal bovine serum + 1% penicillin/streptomycin. The cells were then incubated at 37℃ in a humidified environment with 5% CO_2_. Six-well plates were used to cultivate one million cells per well. In the tests, mRMECs at passages 3–6 were used.

After being treated for an extra hour or 4 h with either FO (60 μg/ml) or SRT1720 (0.5 μM, HY-10532, MedChem Express, Shanghai, PRC), the cells were treated with Ang II (100 nM) for twenty-four hours. Oxidative stress in mRMECs was found using 2',7'-Dichlorodihydrofluorescein diacetate (DCFH-DA, HY-D0940, MedChem Express, Shanghai, PRC) [27, 28].

### Western Blot

Proteinase inhibitor (PMSF, 1:100, Beyotime, Shanghai, PRC) was used to cleave fresh retina tissues and cells in RIPA lysis buffer. A total of 25 μg of protein was separated using SDS-PAGE (10%-12.5%), transferred onto PVDF membranes, and then incubated for an overnight period at 4 °C with the following primary antibodies: anti-SIRT1 (1:800, #27,523, Signalway Antibody), anti-NLRP3 (1:1000, ET1610-93, HUABIO), anti-IL-1β (1:800, WL02257, Wanleibio), and anti-Cleaved-IL-1β (1:800, WL00891, Wanleibio). On the following day, the membranes were with TBST, coated with BeyoECL Star (Beyotime, Shanghai, PRC), and incubated for one hour at room temperature with the secondary antibodies. GAPDH served as the internal control.

### Real-time quantitative polymerase chain reaction (RT-qPCR).

Using Trizol reagent, total RNA was isolated from both fresh and frozen retinal tissues. Next, cDNA is created by reversing the mRNA. Next, RT-qPCR was carried out using a particular primer set and SYBR Green mix. For the *Gapdh* gene, relative gene expression levels were adjusted. Table 1 had a list of the primer.

**Table 1:** The details of primers used in RT-qPCR

Gene	Forward primer (5′-3′)	Reverse primer (5′-3′)
Sirt1	GATACCTTGGAGCAGGTTGC	CTCCACGAACAGCTTCACAA
Nlrp3	TGCCTGTTCTTCCAGACTGGTGA	CACAGCACCCTCATGCCCGG
Tnf	TCACAGACGAATGACTCCAA	GTGCCACTTCATACCAGGAGAA
Nox1	CAGTTATTCATATCATTGCACACCTATTT	CAGAAGCGAGAGATCCATCCA
Nox4	CAGATGTTGGGGCTAGGATTG	GAGTGTTCGGCACATGGGTA
Il1b	TGCCACCTTTTGACAGTGATG	TGATGTGCTGCTGCGAGATT
Il6	TGATGGATGCTACCAAACTGGA	TGTGACTCCAGCTTATCTCTTGG
Gapdh	GGTTGTCTCCTGCGACTTCA	GGTGGTCCAGGGTTTCTTACTC

### Statistics analysis

The mean ± SD is used to show the data. For RT-qPCR and Western Blot analysis, we calculated the data with the mean of housekeeping gene/protein to get the relative expression results. Then, Software called Graph Pad Prism was used to carry out statistical analysis. Dunnett's multiple comparison test and the control group were used after one-way ANOVA for statistical comparison. To compare the two groups, the student's unpaired t test was used. P values less than 0.05 were regarded as statistically significant.

## Results

### Ang II infusion induces SIRT1 reduction and NLRP3 inflammasome activation

**Fig. 1:**
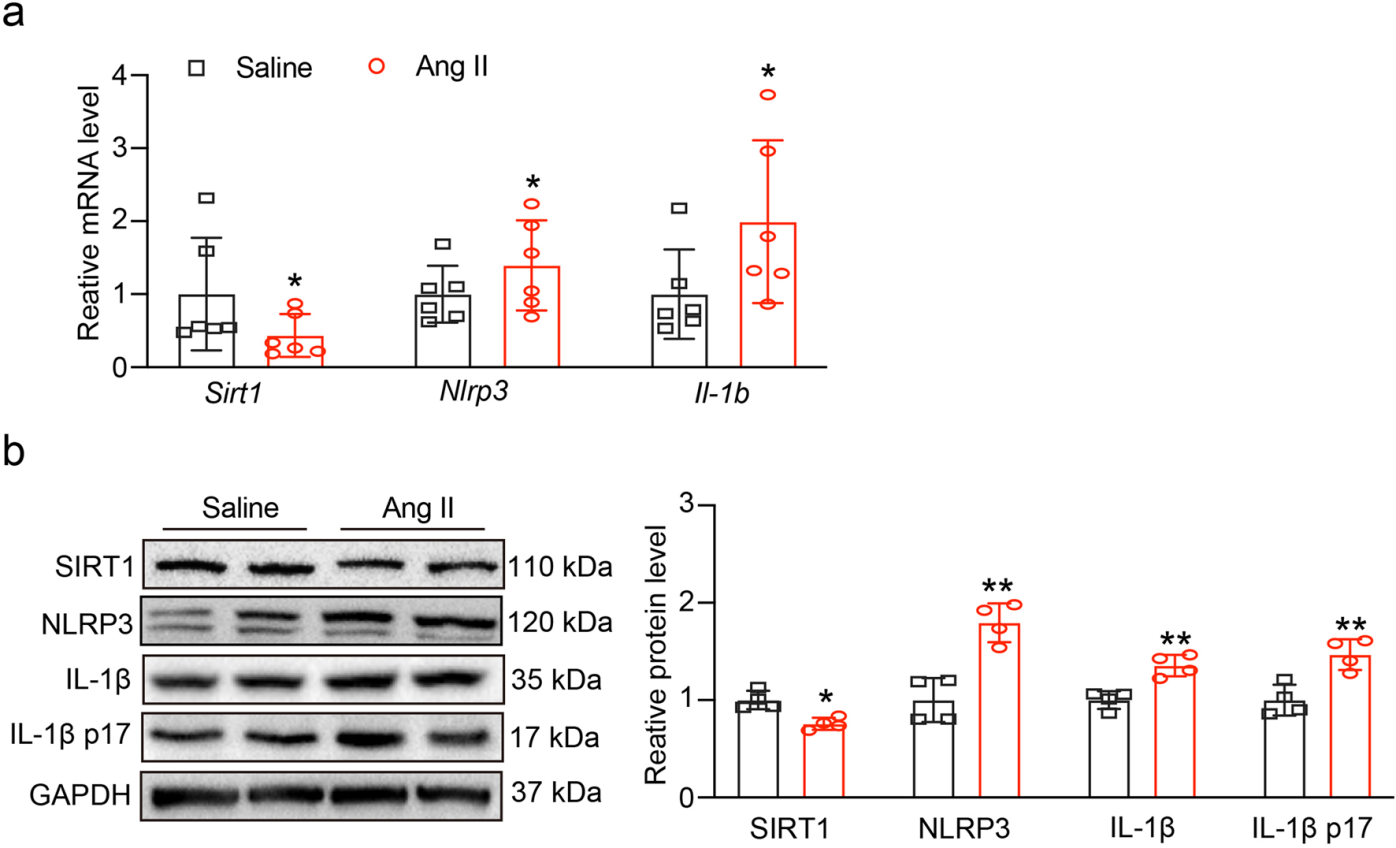
Ang II-induced SIRT1 decrease and overexpression of NLRP3 inflammasome. **A** Mice were infused with Ang II (3000 ng/kg/min) or saline for 3 weeks. qPCR analyses of *Sirt1, Nlrp3* and *Il1b* mRNA in retina (n = 6). **B** The expression of SIRT1, NLRP3, IL-1β and IL-1β p17 protein levels. **C** The quantification of protein expression (n = 4). *P < 0.05, **P < 0.01 vs control

After Ang II (3000 ng/kg/min) or saline infusion for 3 weeks, we evaluated the level of *Sirt1, Nlrp3* and *Il1b* mRNA*.* As shown in Fig. 1a, Ang II infusion decreased the mRNA level of *Sirt1*, while those of *Nlrp3* and *Il1b* were increased significantly. The western blot results showed in Fig. 1b, c, the expression of SIRT1 was also decreased, the expression of NLRP3 and IL-1β and its bioactive form, IL-1β p17 were increased. Those results prompted that SIRT1 and NLRP3 inflammasome might involve in Ang II-induced HR.

### Targeted inhibition of NLRP3 alleviates retinopathy and dysfunction in Ang II-infused mice

**Fig. 2:**
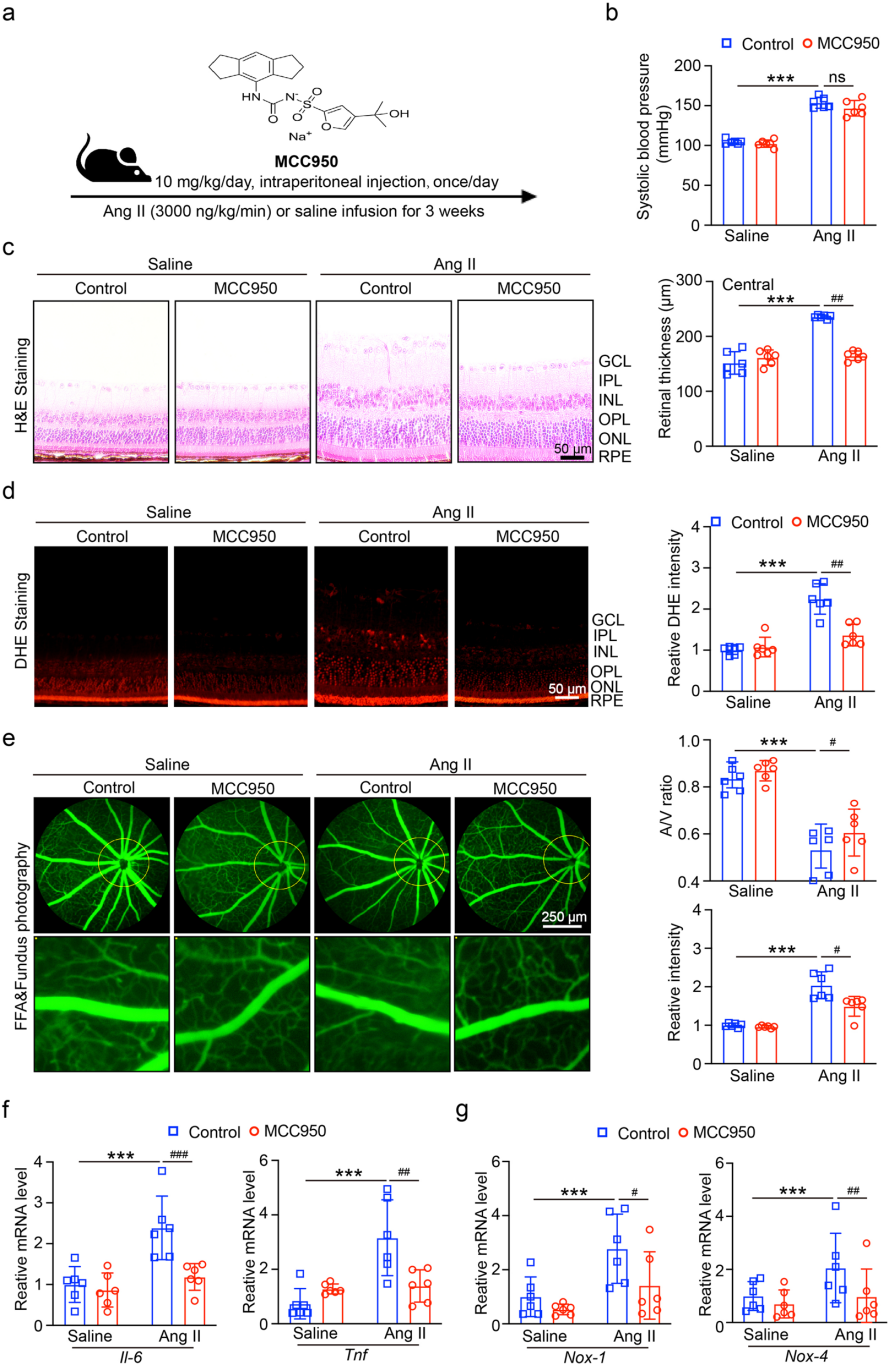
Application of MCC950 decreased Ang II-induced hypertensive retinopathy, ROS production and inflammation. **A** The mice were treated with NLRP3 inhibitor MCC950 (10 mg/kg/day, intraperitoneal injections) one day before Ang II infusion, and then once/day for 3 weeks. **B** SBP of each group was recorded (n = 6). **C** Images of hematoxylin and eosin (H&E) staining of central retinal sections (left), the thickness was quantified (n = 6). **D** Dihydroethidium (DHE) staining of retina in each group (left), the quantification of DHE intensity (n = 6). **E** Typical retinal angiograms and fundus photos (left), these white arrows indicate vascular fluorescein leakage and the corresponding area in the fundus photo. The ratio of retinal arteriovenous and fluorescence intensity was quantified (right; n = 6). **F** qPCR analyses of inflammatory mRNA of *Il6* and *Tnf* (n = 6). **G** qPCR analyses of oxidative stress mRNA of *Nox1* and *Nox4* (n = 6). ***P < 0.0001 vs control; ^ns^*P* > 0.05, ^#^*P* < 0.05, ^##^*P* < 0.01, ^###^*P* < 0.0001 vs Ang II group

To investigate the role of NLRP3 inflammasome in Ang II-induced HR, we treated the mice with NLRP3 inhibitor MCC950 (10 mg/kg/day, intraperitoneal injections) one day before Ang II infusion (Fig. 2a**)**, we found that after treatment with MCC950, the systolic blood pressure (SBP) was not decreased in Ang II-infused mice (Fig. 2b). H&E staining showed that the inhibition of NLRP3 reduced Ang II-induced central retinal thickening (Fig. 2c). We next detected the oxidative stress in each group, the results showed that in Fig. 2d, DHE staining showed the inhibition of NLRP3 had significantly decreased Ang II-induced ROS production. Moreover, Ang II-induced impairment of the retinal arteriolar structure, as indicated by arteriolar narrowing (decreased artery-to-vein (A/V) ratio), tortuosity and exudation, was markedly better in the retinas of MCC950-treated mice than in those of PBS-treated controls (Fig. 2e). RT-qPCR results in Fig. 2f, g showed that NLRP3 inhibition reduced the levels of mRNA expression of NADPH oxidases (*Nox1* and *Nox4*) and inflammatory (*Il6* and *Tnf*) in Ang II-infused mice.

**Fig. 3:**
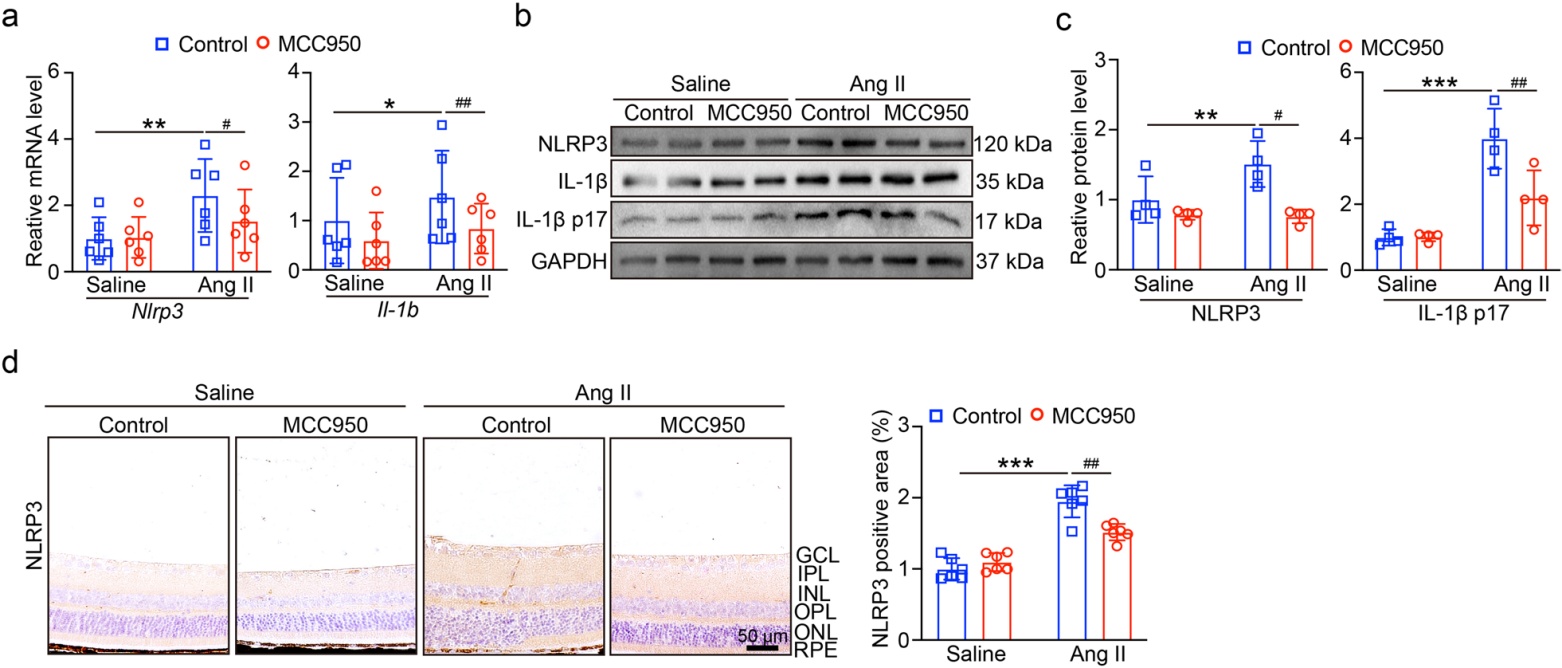
Ang II-induced pyroptosis depend on NLRP3 inflammasome activation. **A** qPCR analyses of *Nlrp3* and *Il1b* mRNA in retina (n = 6). **B** The expression of NLRP3, IL-1β and IL-1β p17 protein levels. **C** The quantification of protein expression (n = 4). **D** Immunohistochemical staining of NLRP3 in retina (left), quantification of NLRP3 positive area (right, n = 6). *P < 0.05, **P < 0.01, ***P < 0.0001 vs control; ^#^*P* < 0.05, ^##^*P* < 0.01 vs Ang II group

In addition, we tested the level of *NLRP3* and *IL-1β* mRNA, the results showed in Fig. 3a, treatment with MCC950 inhibited the level of *Nlrp3* and *Il1b* mRNA in Ang II-infused mice. Similarly, MCC950 significantly suppressed NLRP3 protein levels and IL-1β p17 secretion under Ang II infusion (Fig. 3b, c). The immunohistochemical staining of NLRP3 in Fig. 3d revealed that the retina's ganglia cell layer (GCL), inner plexiform layer (IPL), and inner nuclear layer (INL) were the areas where NLRP3 was most highly expressed. While levels in the outer layers increased following Ang II treatment, NLRP3 expression was significantly suppressed following MCC950 treatment. Above all, the findings indicated that the best option for blocking Ang II-induced HR and NLRP3 inflammsome activation is the NLRP3 protein inhibitor MCC950.

### SIRT1 modulates Ang II-induced injury and NLRP3 inflammasome activation in mRECs

**Fig. 4:**
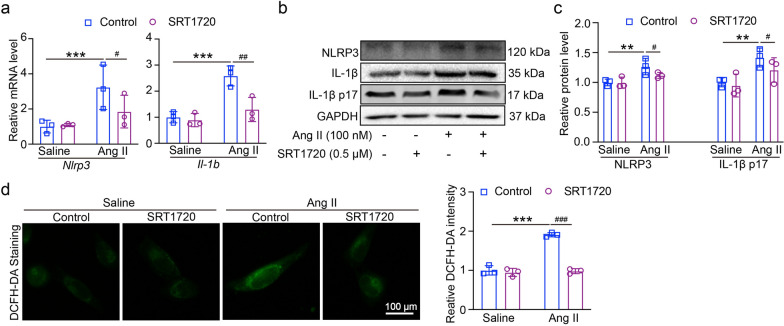
Effect of overexpression of SIRT1 on NLRP3 inflammasome. **A** Mouse retinal vascular endothelial cells (mRECs) were treated with SRT1720 (0.5 μM) for an additional 1 h and then treated with Ang II (100 nM) for 24 h. qPCR analyses of *Nlrp3* and *Il1b* mRNA in mRECs (n = 6). **B** The expression of NLRP3, IL-1β and IL-1β p17 protein levels. **C** The quantification of protein expression (n = 4). (D) DCFH-DA staining of ROS production in cells (left), the quantification of ROS intensity (n = 6). **P < 0.01, ***P < 0.0001 vs control; ^#^*P* < 0.05, ^##^*P* < 0.01, ^###^*P* < 0.0001 vs Ang II group

To assess whether SIRT1 plays a regulatory role in NLRP3 inflammasome activation, we treated the mRECs with SRT1720 (0.5 μM) for an additional 1 h and then treated with Ang II (100 nM) for 24 h. After treatment with SRT1720, the mRNA and protein levels of *Nlrp3* and mRNA of *Il1b* and IL-1β p17 protein expression were both decreased in Ang II-treated cells (Fig. 4a–c). DCFH-DA staining showed that after treatment with SRT1720, the ROS production was reduced in Ang II-treated cells (Fig. 4d). Thus, the upregulation of SIRT1 plays an inhibitory role of Ang II-induced NLRP3 inflammasome activation and ROS production.

### FO enhances SIRT1 expression and reduces NLRP3 activation in Ang II-treated mRECs

**Fig. 5:**
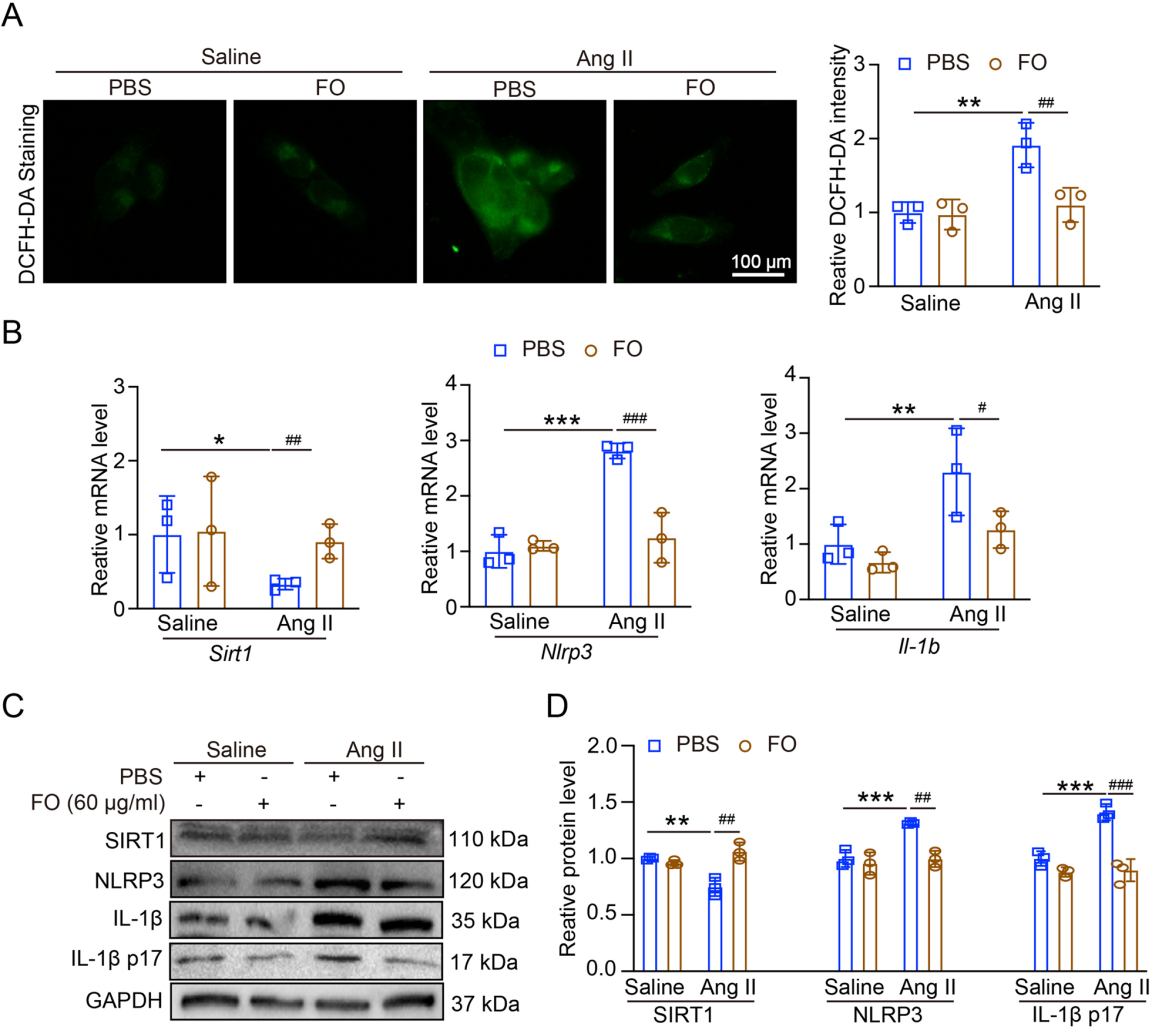
FO upregulated SIRT1 suppressed NLRP3 inflammation activation. **A** mRECs were treated with with FO (60 μg/ml) for an additional 4 h, and then treated with Ang II (100 nM) for 24 h. 2′,7′-Dichlorodihydrofluorescein diacetate (DCFH-DA) staining of ROS production in cells (left), the quantification of ROS intensity (n = 6). **B** qPCR analyses of *Sirt1, Nlrp3* and *Il1b* mRNA in mRECs (n = 6). **C** The expression of SIRT1, NLRP3, IL-1β and IL-1β p17 protein levels. **D** The quantification of protein expression (n = 4). *P < 0.05, **P < 0.01, ***P < 0.0001 vs control; ^#^*P* < 0.05, ^##^*P* < 0.01 vs Ang II group

Fucoidans, which are extracted from various species of brown seaweeds, and have shown a wide spectrum of activities, such as anti-oxidation, anti-aggregation and anti-inflammation [29]. To evaluate the therapeutic effects of FO, we continued to treat the cells with FO (60 μg/ml) for an additional 4 h, and then treated with Ang II (100 nM) for 24 h. The results showed in Fig. 5a, the production of ROS was decreased in Ang II-treated cells after FO treatment. Next, we tested the level of *Sirt1* mRNA, the mRNA level of *Sirt1* was increased by treatment with FO. Similarly, the expression of SIRT1 protein was upregulated after treated with FO (Fig. 5b-d). In addition, we texted the mRNA and protein levels of *Nlrp3*, the expression level of *Nlrp3* mRNA and protein were both decreased in Ang II-treated cells (Fig. 5b-d). Those results suggested that FO could upregulate SIRT1 expression and reduce NLRP3 activation.

### Activation of SIRT1 by FO reduces Ang II-induced retinopathy and NLRP3 inflammasome

**Fig. 6:**
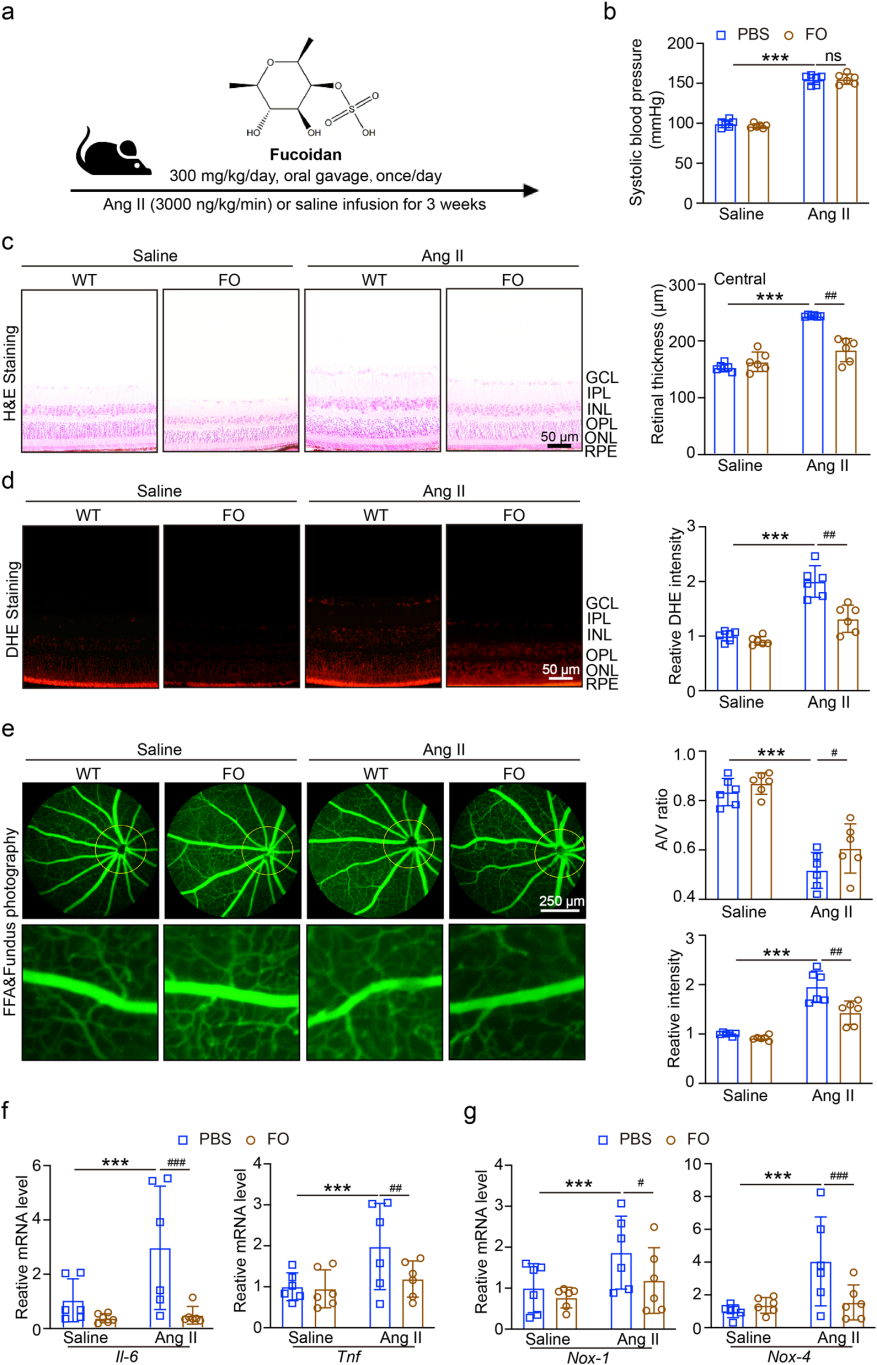
Effects of FO on hypertensive retinopathy and dysfunction in Ang II-infused mice retina. **A** The mice were treated with FO (300 mg/kg/day) one day before Ang II infusion one day before Ang II infusion, and then once/day for 3 weeks. **B** SBP of each group was recorded (n = 6). **C** Images of H&E staining of central retinal sections (left), the thickness was quantified (n = 6). **D** DHE staining of retina in each group (left), the quantification of DHE intensity (n = 6). **E** Typical retinal angiograms and fundus photos (left), these white arrows indicate vascular fluorescein leakage and the corresponding area in the fundus photo. The ratio of retinal arteriovenous and fluorescence intensity was quantified (right; n = 6). **F** qPCR analyses of inflammatory mRNA of *Il6* and *Tnf* (n = 6). **G** qPCR analyses of oxidative stress mRNA of *Nox1* and *Nox4* (n = 6)

We gave the mice with FO (300 mg/kg/day) one day before Ang II infusion (Fig. 6a). We found that after treatment with FO, the SBP was not decreased in Ang II-infused mice (Fig. 6b). H&E staining showed that FO treatment decreased Ang II-induced central retinal thickening (Fig. 6c). We next detected the oxidative stress in each group, the results showed that in Fig. 6d, DHE staining showed treatment with FO had significantly inhibited Ang II-induced ROS production. RT-qPCR results in Fig. 6e, f showed that FO reduced the levels of mRNA expression of *Nox1, Nox4, Il6* and *Tnf* in Ang II-infused mice.

**Fig. 7:**
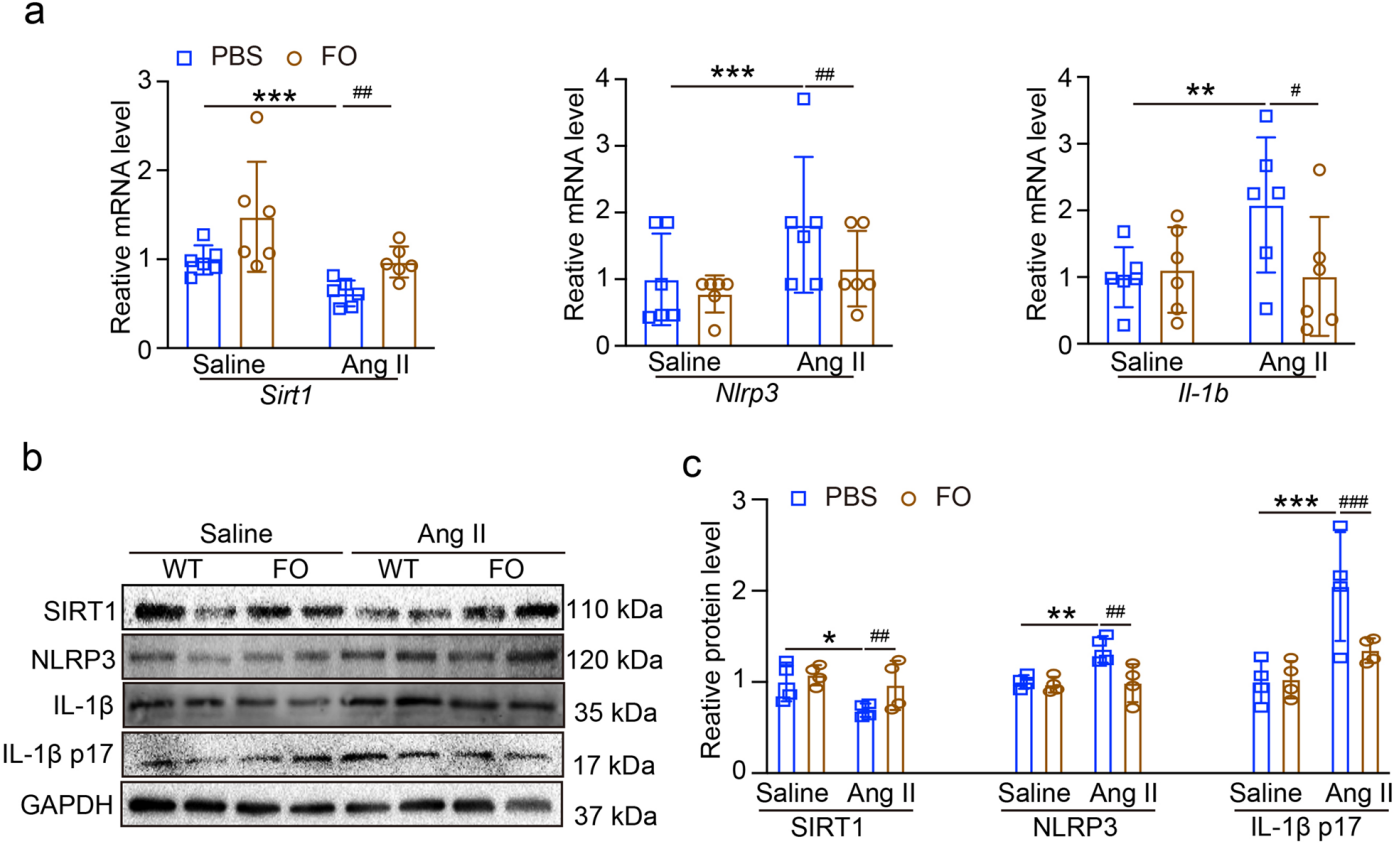
Treatment with FO on regulating SIRT1/NLRP3 pathway. **A** qPCR analyses of *Sirt1, Nlrp3* and *Il1b* mRNA in retina (n = 6). **B** The expression of SIRT1, NLRP3, IL-1β and IL-1β p17 protein levels. **C** The quantification of protein expression (n = 4). *P < 0.05, **P < 0.01, ***P < 0.0001 vs control; ^#^*P* < 0.05, ^##^*P* < 0.01, ^###^*P* < 0.0001 vs Ang II group

Next, we tested the level of *Sirt1, Nlrp3* and *Il1b* mRNA, the results showed in Fig. 7a, treatment with FO increased the level of *Sirt1* mRNA, and inhibited the level of *Nlrp3* and *Il1b* mRNA in Ang II-infused mice. Similarly, FO significantly upregulated SIRT1 and suppressed NLRP3 protein levels and IL-1β p17 secretion under Ang II infusion (Fig. 7b, c).

## Discussion

It is well recognized that hypertension increases the risk of a number of illnesses, including heart failure, renal failure, stroke, disability, and early death [30]. A number of pathophysiological changes brought on by hypertension may harm the retinal, choroidal, and optic nerve circulations, resulting in retinopathy, choroidopathy, and optic neuropathy, in that order, in the eyes [2, 31, 32]. During the first phase, the retinal arterioles undergo vasoconstriction and localized vasospasm in response to high blood pressure. The local autoregulatory systems responsible for optimizing blood flow are the cause of the vasospasm. The clinical manifestation of these occurrences is a reduction in the normal arteries to vein ratio, which indicates either localized or global constriction of the retinal arteries. Over time, high blood pressure causes structural alterations in the arterial wall, including hyaline degeneration, mediawall hyperplasia, endothelial damage, and intimal thickening. This phase causes the vessel walls' focused or diffuse light response to be emphasized, as well as a shift in arteriovenous crossing or nicking [33–35].

**Fig. 8:**
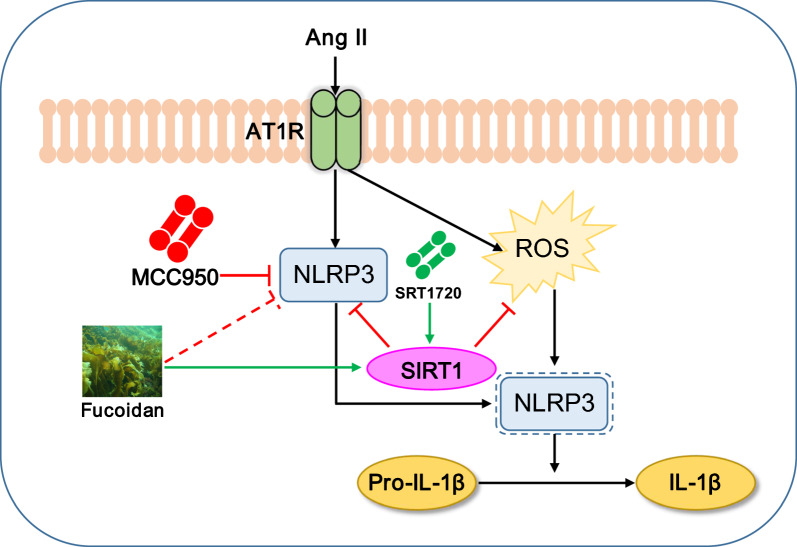
A working model of the mechanism by SIRT1/NLRP3 in Ang II-induced hypertensive retinopathy. Ang II infusion induced hypertensive retinopathy, ROS production, SIRT1 reduction and NLRP3 inflammasome activation. FO could rescue those reactions. SIRT1/NLRP3 might by a new sight of hypertensive retinopathy and dysfunction and FO might be used as an agent to protect against Ang II-induced hypertensive retinopathy

Numerous investigations have shown that inflammation is a significant factor in hypertensive retinal vascular damage and the retinopathy that follows [33]. Our findings show that Ang II-induced retinal lesions in mRECs and mice were caused by inflammation and pyroptosis linked to the NLRP3 inflammasome, and that SIRT1 is an upstream negative regulator that blocks the NLRP3 inflammasome pathway. These results may have therapeutic implications since they point to possible mechanism-based medication approaches for the treatment of HR (Fig. 8).

In many disorders, the NLRP3 inflammasome is important in controlling the immune system's inflammatory reactions [5]. According to recent research, individuals with hypertension consistently have elevated plasma levels of NLRP3 [36]. The pro-inflammatory kind of cell death known as pyroptosis, which is brought about by NLRP3 activation, exacerbates the inflammatory response by causing the release of IL-1β and other pro-inflammatory intracellular components [37]. Vascular dysfunction and pro-inflammatory cytokine levels may be correlated [38]. Moreover, cytokine levels, including as *Tnf, Il6*, and *Il1b*, are downregulated when the NLRP3 inflammasome is inhibited [39]. It is advantageous to inhibit the NLRP3 inflammasome in order to lessen inflammation and the pathological alterations that follow from inflammation [7].

Our research revealed that Ang II infusion may increase the expression of NLRP3 and reduce the level of SIRT1 (Fig. 1); these findings showed that Ang II-induced hypertensive retinopathy and dysfunction may involve both SIRT1 and NLRP3. Next, we administered MCC950, an NLRP3 inhibitor, to the mice. Following MCC950 therapy, we observed that NLRP3 and IL-1β expression were suppressed, and that in Ang II-infused animals, NLRP3 suppression reduced retinopathy and dysfunction (Figs. 2, 3). In order to examine SIRT1's function in the Ang II-induced NLRP3 inflammasome and HR, we administered SIRT1 agonist, SRT1720, to the mRECs. The data in Fig. 4 demonstrated how Ang II-induced NLRP3 inflammasome activation and ROS generation are inhibited by SIRT1 overexpression. As a sensor and defender of the redox environment, SIRT1 is involved in the control of cell survival, apoptosis, and inflammation [40]. It is a NAD-dependent deacetylase that controls how proteins function through lysine residue deacetylation. According to a publication, SIRT1 prevents NLRP3 inflammasome-induced IL-1β production, therefore shielding mesenchymal stem cells from radiation damage [41]. SIRT1 may also deacetylate NF-κB to promote the suppression of NLRP3 inflammasome activation [7]. Inflammation and cell pyroptosis linked to the NLRP3 inflammasome are negatively regulated by SIRT1, and this has an impact on avoiding Ang II-induced HR and malfunction. Furthermore, p53, another transcription factor that targets apoptosis-associated speck-like protein containing a CARD (ASC), which is necessary for NLRP3 inflammasome assembly, was affected by SIRT1's diverse deacetylase activity [42]. Thus, it is plausible that SIRT1 acted as an upstream regulator of the activation of the NLRP3 inflammasome produced by Ang II in conjunction with the current investigation. SIRT1 overexpression could significantly decrease the inflammasome activation. In our present study, we found FO could inhibit apoptosis and improve cardiac remodeling by inhibiting tumor suppressor protein (p53) transcriptional activation through ubiquitin-specific protease (USP22)—SIRT1 [24]. FO mainly extracted from brown algae is a fucose-enriched sulfated polysaccharide, and it has been widely used as a dietary supplement and health food due to its numerous beneficial effects, including anti-inflammatory, anticancer, and antidiabetic activities [18]. Recent studies have found that FO reduced secretion and expression of vascular endothelial growth factor in the retinal pigment epithelium and reduced angiogenesis in vitro [43]. Fucoidan is currently considered a functional food, but is also investigated in clinical trials [44]. Its effects have been studied not only in vitro, but also in animal and human studies, were it exhibits an excellent toxic profile. While its oral availability is under debate, recent studies indicate a possible absorption of fucoidan by the gastrointestinal tract, which would render an oral application an attractive alternative to intravitreal injections [29]. In our study, we found FO enhanced SIRT1 expression and reduced NLRP3 activation and retinopathy and dysfunction in Ang II-treated mice and mRECs (Fig. 5–7). This approach provides a potential targeted strategy to treat HR and dysfunction. But we have some limitations, one potential drawback is the study's applicability to human populations, which could be addressed by discussing any known similarities and differences in the SIRT1 and NLRP3 inflammasome pathways between mice and humans. Future research could focus on the exploration of the SIRT1/NLRP3 pathway in other models of hypertensive organ damage, and translation into clinical research. The clinical significance would be more compelling if it included functional endpoints that mirror human disease, such as vision acuity or electrophysiological assessments of retinal function.

## Conclusion

This study has shown that Ang II-infusion caused HR and dysfunction through altering SIRT1 decrease and NLRP3 inflammmasome activation overexpression. Here, we discovered that FO therapy decreased NLRP3 activation, retinopathy, and dysfunction while increasing SIRT1 expression. This method offers a possible focused treatment plan for dysfunction in HR.

## Data Availability

All data generated or analysed during this study are included in this published article.

## Abbreviations

SIRT1: Sirtuin 1
Ang II: Angiotensin II
NLRP3: NOD-like receptor thermal protein domain associated protein 3
FO: Fucoidan
mRECs: Mouse retinal vascular endothelial cells
H&E: Hematoxylin eosin
DHE: Dihydroethidiumto
RT-qPCR: Real-time quantitative polymerase chain reaction
HR: Hypertensive retinopathy
NLRPs: NOD-like receptor thermal protein domain associated proteins
AIM2: Absent in melanoma 2
NF-κB: Nuclear factor kappa B
IL-1β: Interleukin-1β
ROS: Reactive oxygen species
AMPK: AMP-activated catalytic subunit alpha 1
PPARα: Peroxisome proliferator activated receptor alpha
PGC-1α: Peroxisome proliferative activated receptor, gamma, coactivator 1 alpha
WT: Wild type
H-DMEM: High glucose-dulbecco’s modified eagle medium
DCFH-DA: 2′,7′-Dichlorodihydrofluorescein diacetate
PVDF: Polyvinylidene fluoride
GCL: Ganglia cell layer
IPL: Inner plexiform layer
INL: Inner nuclear layer
ASC: Apoptosis-associated speck-like protein containing a CARD
p53: Tumor suppressor protein
